# High sensitivity fiber optic temperature sensor composed of two parallel FPI and enhanced harmonic Vernier effect

**DOI:** 10.1038/s41598-025-96809-7

**Published:** 2025-04-28

**Authors:** Huiling Huang, Chao Jiang, Xiaoshan Guo, Simei Sun, Tingshui Cao, Long Zhang, Tianqi Yan

**Affiliations:** Hubei Key Laboratory of Optoelectronic Conversion Materials and Devices, Hubei Engineering Research Center for Micronano Optoelectronic Devices and Integration, College of Physics and Electronic Science, Hubei Normal University, Huangshi, 435002 Hubei People’s Republic of China

**Keywords:** Fiber optic temperature sensor, Ceramic ferrule, Fabry–Perot interferometer, Polydimethylsiloxane, Harmonic Vernier effect, Vernier effect, Applied optics, Optical techniques, Optics and photonics

## Abstract

A high-sensitivity fiber optic temperature sensor based on the enhanced harmonic Vernier effect (HVE) is proposed, which consists of two Fabry–Perot interferometers (FPI) that are sensitive to temperature and connected in parallel. FPI_1_ is a polydimethylsiloxane (PDMS) cavity formed by filling a ceramic ferrule with PDMS, and FPI_2_ is an air-cavity formed by inserting a single-mode fiber into a ceramic ferrule coated with PDMS film on the end face. FPI_1_ and FPI_2_ have opposite temperature responses and an approximate 2-fold free spectral range (FSR) relationship. As the temperature rises, the interference spectrum of FPI_1_ gradually red-shifts, while the interference spectrum of FPI_2_ gradually blue-shifts, resulting in an enhanced HVE. Its temperature sensitivity is much higher than that of a single FPI, and the amplification rate is significantly higher than that of ordinary Vernier effect. Two enhanced HVE sensors S_1_ and S_2_ are developed using this method, but there is a certain difference in their FSR detuning. The experimental results reveal that within the temperature range of 30–35 °C, the temperature sensitivity of S_1_ and S_2_ reach − 44.39 nm/°C and − 23.14 nm/°C, respectively. Both S_1_ and S_2_ have extremely high temperature sensitivity, but FSR detuning has a significant impact on sensitivity amplification. Additionally, the proposed enhanced HVE sensor has good repeatability and stability in measuring temperature.

## Introduction

Temperature is a common and important physical quantity, and in fields such as petroleum and petrochemicals, food engineering, medicine and healthcare, and earth resource exploration, precise measurement of temperature changes is often required. Compared with other types of temperature sensors, fiber optic temperature sensors have the following advantages, including compact size, fast response, no electromagnetic interference, safety and no pollution to the environment, as well as remote and distributed sensing capabilities^1–10^. At present, the fiber optic sensor structures used for temperature measurement mainly include the following types: fiber optic grating^1^, fiber surface plasmon resonance (SPR)^2,3^, bow-shaped fiber structure^4^, S-taper fiber mode interferometer^5^, Mach–Zehnder interferometer (MZI)^6,7^, Fabry–Perot interferometers (FPI)^8,9^, Sagnac interferometers (SI)^10^, etc. Although these sensors have simple structures, easy to manufacture, and strong robustness, the sensitivity of some structures is still relatively low.

The sensitivity of fiber optic sensors is one of their most critical parameters, which affects the resolution, accuracy, stability, dynamic range, and response time of the sensor. It is also a key parameter for us to design and select sensors. In practical industrial applications, the demand for high-sensitivity sensors is increasing day by day. These high-sensitivity sensors can improve the effectiveness and efficiency of sensing measurements. Therefore, seeking new structures to further improve the sensitivity of sensors is one of the goals pursued by people. Since the optical Vernier effect (VE) was first proposed for sensitivity amplification applications in sensors in 2014^11^, different fiber optic VE temperature sensor structures have been explored^12^. Especially the optical fiber VE temperature sensors made using FPI are favored by everyone due to its simple design, easy manufacturing, stable structure, high resolution and sensitivity^13–17^. On the basis of VE, an enhanced VE has been developed, which can further amplify the sensitivity of sensors. At this point, both interferometers are sensitive to the measured object, but the sensitivity signs are opposite. They are both sensing cavities and reference cavities for each other. Several temperature sensors based on enhanced VE have also been proposed^18–22^. The VE temperature sensor has achieved extremely high sensitivity, but there is also a defect in that two interferometers require precise matching to produce a Vernier effect.

In 2019, Gomes et al. first proposed the harmonic Vernier effect (HVE), which was formed by increasing the optical path length (OPL) of one interferometer to *i* times the OPL of the second interferometer (where *i* is the harmonic order, usually a positive integer)^23^. HVE does not require strict FSR matching conditions like traditional VE. It allows for greater manufacturing tolerances without sacrificing sensitivity^23^. Moreover, HVE examines the variation of internal envelope intersections with environmental parameters, thus possessing higher measurement accuracy. In recent years, temperature sensors based on HVE have also attracted people’s attention, and a large number of HVE temperature sensors have been proposed^24–33^. These sensors are prone to Vernier effect, reducing the difficulty of production and achieving a maximum sensitivity of − 99.66 nm/°C^33^.

In this study, we will combine the enhanced VE with the HVE to reduce the difficulty of sensor fabrication while achieving high sensitivity. Two FPIs were constructed using single-mode fiber (SMF), ceramic ferrule, polydimethylsiloxane (PDMS), etc. Among them, FPI_1_ is temperature sensitive and its sensitivity is positive, while FPI_2_ is temperature sensitive and its sensitivity is negative. When their free spectral range (FSR) is approximately twice relationship, their parallel connection will form an enhanced HVE sensor. We have developed two enhanced HVE sensors S_1_ and S_2_ using this method, but there is a certain difference in their FSR detuning. We conducted experimental explorations on S_1_ and S_2_ and found that they have extremely high temperature sensitivity, and the FSR detuning has a significant impact on sensitivity amplification. Additionally, the proposed enhanced HVE sensor has good stability and repeatability, low preparation cost, and very good application prospects.

## Sensor configuration and sensing principle

### Principle of the sensor

**Fig. 1:**
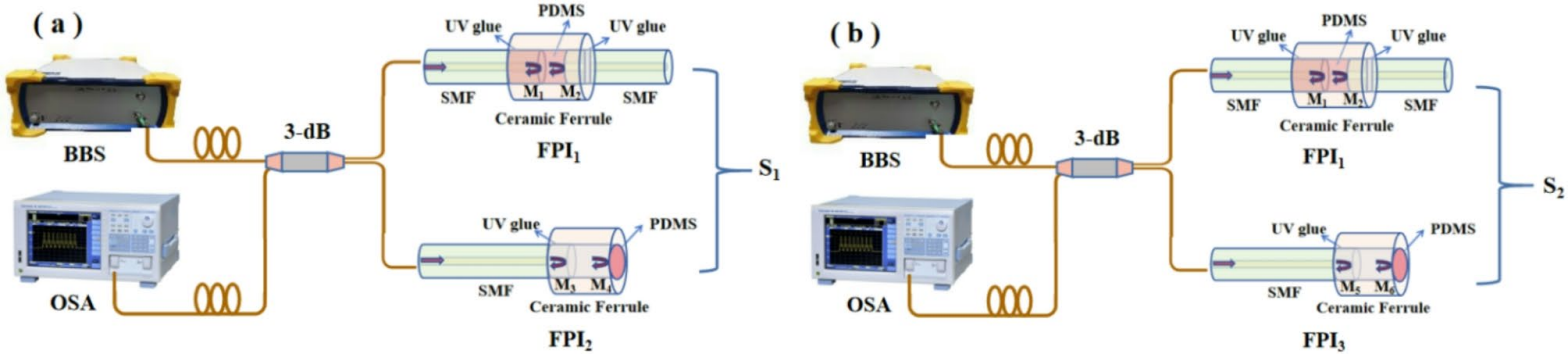
Schematic of two sensing head of the proposed temperature sensor.

Figure 1 shows the schematic diagram and light path of two designed temperature sensors S_1_ and S_2_. In Fig. 1, S_1_ is composed of FPI_1_ and FPI_2_ connected in parallel, and S_2_ is composed of FPI_1_ and FPI_3_ connected in parallel. The materials used in this sensor mainly include standard single-mode fiber (SMF), ceramic ferrules, PDMS, etc. FPI_1_ is generated by the interference of light from two reflecting surfaces of M_1_ and M_2_. FPI_2_ (or FPI_3_) is produced by the interference of light from two reflecting surfaces of M_3_ and M_4_. FPI_3_ is produced by the interference of light from two reflecting surfaces of M_5_ and M_6_. Due to PDMS being an excellent thermosensitive material, FPI_1_, FPI_2_, FPI_3_ composed of PDMS are temperature sensitive, therefore, S_1_ and S_2_ are also extremely sensitive to temperature.

In Fig. 1, based on the FPI theory, the dip wavelengths and FSRs of the reflection spectra of FPI_1_, FPI_2_, and FPI_3_ can be derived as follows^18^:

1 $${\lambda _1}=\frac{{4{n_1}{L_1}}}{{2k+1}},\quad {\lambda _2}=\frac{{4{n_2}{L_2}}}{{2k+1}},\quad {\lambda _3}=\frac{{4{n_3}{L_3}}}{{2k+1}},\quad (k=1,\;2,\;3, \ldots )$$

2 $$FS{R_1}=\frac{{{\lambda ^2}}}{{2{n_1}{L_1}}},\quad FS{R_2}=\frac{{{\lambda ^2}}}{{2{n_2}{L_2}}},\quad FS{R_3}=\frac{{{\lambda ^2}}}{{2{n_3}{L_3}}}$$

where *n*_1_ is the refractive index (RI) of PDMS of FPI_1_, *n*_2_ is the air RI of FPI_3_, and *n*_3_ is the air RI of FPI_3_. *L*_1_ is the length of PDMS cavity of FPI_1_, *L*_2_ is the air-cavity length of FPI_2_, and *L*_3_ is the air-cavity length of FPI_3_.

FPI_1_ is a PDMS cavity, therefore the temperature sensitivity of FPI_1_ can be expressed as follows:

3 $${S_1}=\frac{{\Delta {\lambda _1}}}{{\Delta T}}={\lambda _1}\left( {\frac{{\Delta {n_1}}}{{{n_1}\Delta T}}+\frac{{\Delta {L_1}}}{{{L_1}\Delta T}}} \right)={\lambda _1}({\alpha _1}+{\beta _1})$$

where ${\alpha _1}$ and ${\beta _1}$ are the thermal optical coefficient and thermal expansion coefficient of PDMS, respectively. Usually, ${\alpha _1}$ is negative and ${\beta _1}$ is positive, but the absolute value ${\beta _1}$ is greater than that of ${\alpha _1}$. Therefore, S_1_ is a positive number.

FPI_2_ (or FPI_3_) is air-cavity with the PDMS film reflecting surface. When the temperature changes, the air RI in the air-cavity is basically not affected, and the cavity length is affected by the change of PDMS film. Therefore, the temperature sensitivity of FPI_2_ (or FPI_3_) can be expressed as follows:

4 $${S_2}=\frac{{\Delta {\lambda _2}}}{{\Delta T}}={\lambda _2}\left( {\frac{{\Delta {n_2}}}{{{n_2}\Delta T}}+\frac{{\Delta {L_2}}}{{{L_2}\Delta T}}} \right) \approx {\lambda _1} \cdot \frac{{\Delta {L_2}}}{{{L_2}\Delta T}}$$

In FPI_2_ (or FPI_3_), the PDMS film coated on the end face of the ceramic ferrule is relatively uniform. The increase in ambient temperature causes the PDMS film to expand to both sides, resulting in a shorter cavity length of the air-cavity of FPI_2_. Therefore, S_2_ is negative number.

Supposing FPI_s_ is a sensing unit and FPI_r_ is a reference unit, their parallel connection will produce the traditional Vernier effect (TVE) when their FSRs are similar. The outer-envelope FSR of TVE can be expressed as:

5 $$FS{R_{en}}=\frac{{FS{R_r} \times FS{R_s}}}{{\left| {FS{R_r} - FS{R_s}} \right|}}$$

wherein $FS{R_s}$ and $FS{R_r}$ represent the FSR of FPI_s_ and FPI_r_ respectively.

The relationship between the magnification factor (M) of TVE and $FS{R_{en}}$ and $FS{R_s}$ is as follows

6 $$m=\frac{{FS{R_{en}}}}{{FS{R_s}}}$$

When FPI_1_, FPI_2_, and FPI_3_ are used as sensing interferometers respectively, their magnification factors for sensitivity are obtained as follows:

7 $${m_1}=\frac{{FS{R_{en}}}}{{FS{R_1}}},\quad {m_2}=\frac{{FS{R_{en}}}}{{FS{R_2}}},\quad {m_3}=\frac{{FS{R_{en}}}}{{FS{R_3}}}$$

If the $FS{R_s}$ of FPI_s_ is the *i* (*i* is integer) times of the $FS{R_r}$ of FPI_r_, the high-order harmonic Vernier effect (HVE) can be generated. At this time, the outer-envelope $FSR_{{en}}^{i}$ of HVE spectrum can be expressed as

8 $$FSR_{{en}}^{i}=\frac{{FS{R_r} \times FS{R_s}}}{{\left| {FS{R_s} - (i+1)FS{R_r}} \right|}}$$

In addition, the inner-envelope $FSR_{{in{\text{-}}en}}^{i}$ of the HVE spectrum can be expressed as

9 $$FSR_{{in{\text{-}}en}}^{i}=\frac{{(i+1)FS{R_r} \times FS{R_s}}}{{\left| {FS{R_s} - (i+1)FS{R_r}} \right|}}=(i+1)FSR_{{en}}^{i}$$

As can be seen, the FSR of the inner envelope is (*i* + 1) times that of the outer envelope, and the magnification factor of the HVE can be expressed as

10 $${m^i}=\frac{{FSR_{{in{\text{-}}en}}^{i}}}{{FS{R_s}}}=(i+1)m$$

If two FPIs are sensitive to the measured physical quantity and their sensitivity signs are opposite, when they are connected in parallel, they serve as reference cavities for each other and can be used together as sensing cavities to amplify sensitivity. They simultaneously amplify the sensitivity of the sensor, which is known as the enhanced Vernier effect. As can be seen from the previous analysis, the sensitivity of FPI_1_ is S_1_ (> 0) and the sensitivity magnification factor is *m*_1_, and the sensitivity of FPI_2_ is S_2_ (< 0) and the sensitivity magnification factor is *m*_2_, therefore assuming that the sensitivity of the enhanced Vernier effect they form can be expressed as^24–27^:

11 $${S_{en}}={m_1}\left| {{S_1}} \right|+{m_2}\left| {{S_2}} \right|$$

Additionally, FPI_1_ and FPI_2_ also form a first-order HVE, therefore FPI_1_ and FPI_2_ form a first-order enhanced HVE sensor (S_1_). Since the magnification factor of the harmonic Vernier being (*i* + 1) times that of the conventional Vernier, the sensitivity ($S_{{e{n_1}}}^{1}$) of the intersection of the internal envelope of S_1_ can be expressed as:

12 $$S_{{e{n_1}}}^{1}=2({m_1}\left| {{S_1}} \right|+{m_2}\left| {{S_2}} \right|)$$

Similarly, FPI_1_ and FPI_3_ also form a first-order enhanced HVE sensor (S_2_), and the sensitivity ($S_{{e{n_2}}}^{1}$) of S_2_ is be expressed as:

13 $$S_{{e{n_2}}}^{1}=2({m_1}\left| {{S_1}} \right|+{m_3}\left| {{S_3}} \right|)$$

### Fabrication and spectrum of the sensor

The key to manufacturing the two sensors S_1_ and S_2_ is to manufacture FPI_1_, FPI_2_, and FPI_3_ separately according to the design requirements. The materials used in this sensor mainly include SMF, ceramic ferrules, PDMS, etc. SMF is produced by Wuhan Changfei optical fiber and cable company, and its core/cladding diameter is 9/125 µm. The end face of ceramic ferrule is planar (FC/PC), and its inner-diameter is 126 μm. The SMF with stripped coating can be accurately inserted into the ceramic ferrule. PDMS is a typical thermosensitive material with high thermal expansion coefficient and thermal optical coefficient. But it is insensitive to humidity and can be considered a hydrophobic material.

The fabrication method of FPI_1_ can be summed up as follows. In the first stage, we cut flat the SMF end face and place it into the ceramic ferrule’s end face hole. After applying UV glue, we expose it to a UV lamp for one to two min to allow the glue to cure. Then the ceramic ferrule with SMF inserted inside into is immersed into PDMS liquid (S184-A and S184-B at a ratio of 10:01), allowing PDMS to be completely sucked into the holes inside the ceramic ferrule. In the second stage, a precision displacement platform was used to slowly insert another SMF into the ceramic ferrule. Optical spectrum analyzer (OSA) and broadband light source (BBS) were used to track the structure’s reflectance spectrum throughout the interim. Another SMF was firmly adhered to the ceramic ferrule’s tail handle using UV glue if the structure’s fringe contrast and FSR satisfied the necessary criteria. After the UV glue is exposed with the UV lamp and FPI_1_ is finished.

The manufacturing process of FPI_2_ and FPI_3_ is the same, only their F-P cavity lengths are different. Their manufacturing process can be described as follows. In the first stage, the ceramic ferrule’s end face is covered with the PDMS solution sealing the tiny hole. In the second stage, once the PDMS solution has fully dried, the SMF end is cut flat and then the SMF is inserted into the ceramic ferrule utilizing a precision displacement platform. An OSA is used to track the structure’s real-time reflection spectrum. If the spectral FSR and the fringe contrast meet the requirements, SMF is fixed on the tail handle of the ceramic ferrule with UV glue, and irradiate it with UV lamp for 1–2 min to cure the UV glue.

**Fig. 2:**
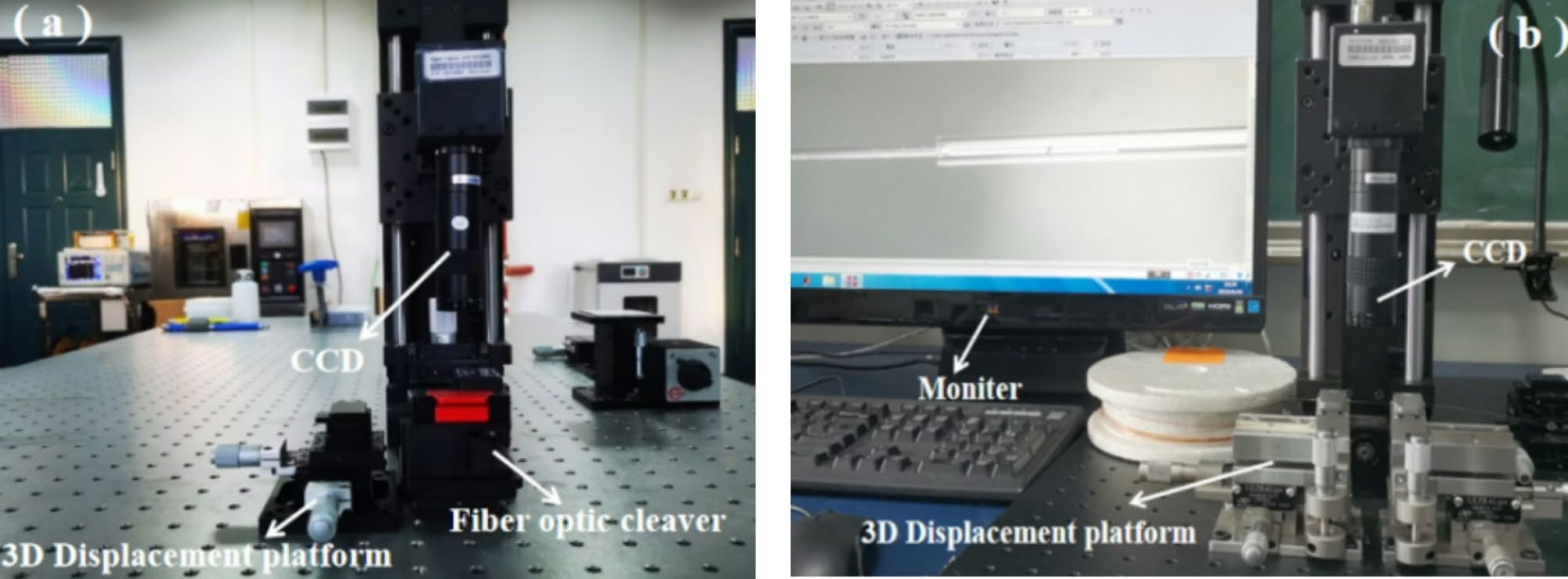
(**a**) Precision cutting system for optical fiber length, (**b**) accurate alignment system for optical fiber and ceramic ferrule.

The key to completing the three FPI production processes is the fiber optic precision cutting and alignment system. We have established a fiber optic precision cutting system and a fiber optic precision alignment system in the laboratory, as shown in Fig. 2. The main components of the system include fiber optic cleaver, CCD imaging systems, 3D precision displacement platforms, monitoring computers, BBS, and OSA. By using CCD and OSA in the system to observe and adjust the size of the SMF inserted into the ceramic ferrule in real time, the length of the F-P cavity can be controlled and the two sections of the SMF can be fully aligned. To obtain the desired FPI structure.

**Fig. 3:**
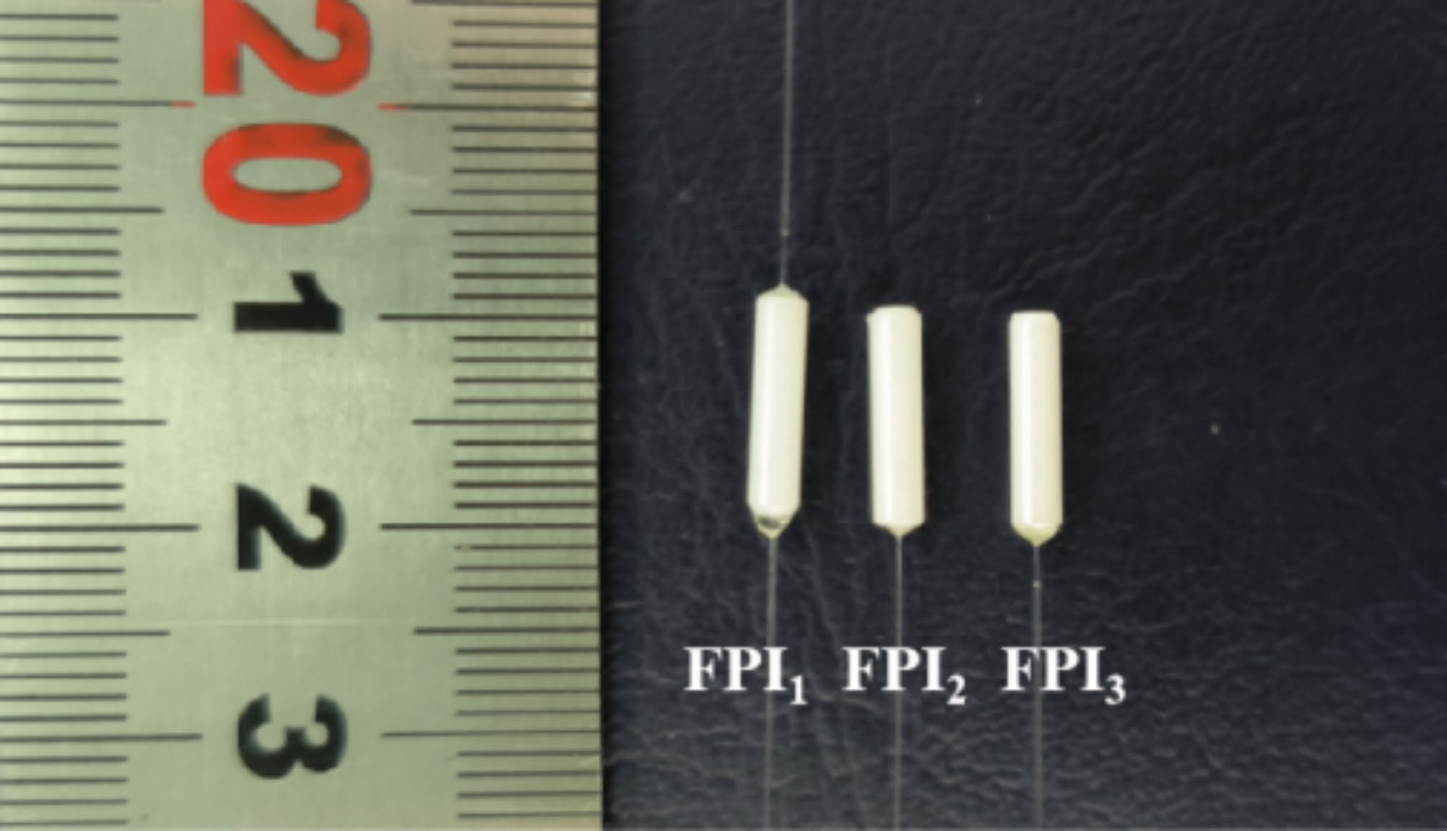
Physical images of FPI_1_, FPI_2_, and FPI_3_.

Figure 3 shows the real images of FPI_1_, FPI_2_, and FPI_3_ captured by a camera. Obviously, the length of the ceramic ferrules in the three FPIs is approximately 10 mm. The designed sensor is composed of ceramic ferrules, SMF, and PDMS materials, with a particularly small volume, low cost, and relatively sturdy structure. We measured the thickness of PDMS films in FPI_2_ and FPI_3_ using a metallographic microscope, which is approximately 23 μm and 20 μm respectively. Due to the thin film and the high amount of dust and dirt on the outer surface in contact with air, PDMS film can be equivalent to a single reflective surface M_4_ (or M_6_), which together with the reflective surface M_3_ (or M_5_) forms FPI_2_ (or FPI_3_). Since the F-P cavity lengths of the three FPIs are sealed inside the ceramic ferrules, we cannot measure their lengths. However, we can obtain the FSRs of the three FPIs during the manufacturing process. Based on Eq. (2), we can estimate the three F-P cavity lengths L_1_, L_2_, and L_3_ of FPI_1_, FPI_2_, and FPI_3_ to be approximately 159 μm, 100 μm, and 97 μm, respectively.

**Fig. 4:**
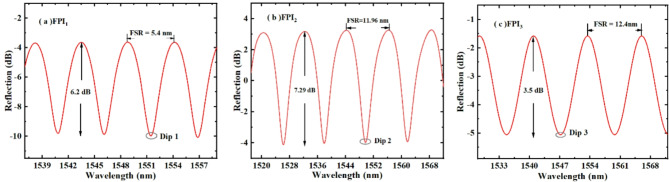
Spectral diagrams of FPI_1_, FPI_2_, and FPI_3_.

We connected FPI_1_, FPI_2_, and FPI_3_ to BBS and OSA respectively through an optical circulator, and measured their spectra as shown in Fig. 4. Obviously, the spectra of FPI_1_, FPI_2_, and FPI_3_ are both approximate sine waves, and their spectral intensities are 6.2 dB, 7.3 dB, and 3.5 dB, respectively. The FSRs of FPI_1_, FPI_2_, and FPI_3_ are 5.4 nm, 11.96 nm, and 12.4 nm, respectively, around 1550 nm. It can be seen that the FSR of FPI_2_ and FPI_3_ is about 2 times that of FPI_1_. Therefore, when they are connected in parallel respectively, the first-order HVE can be formed.

**Fig. 5:**
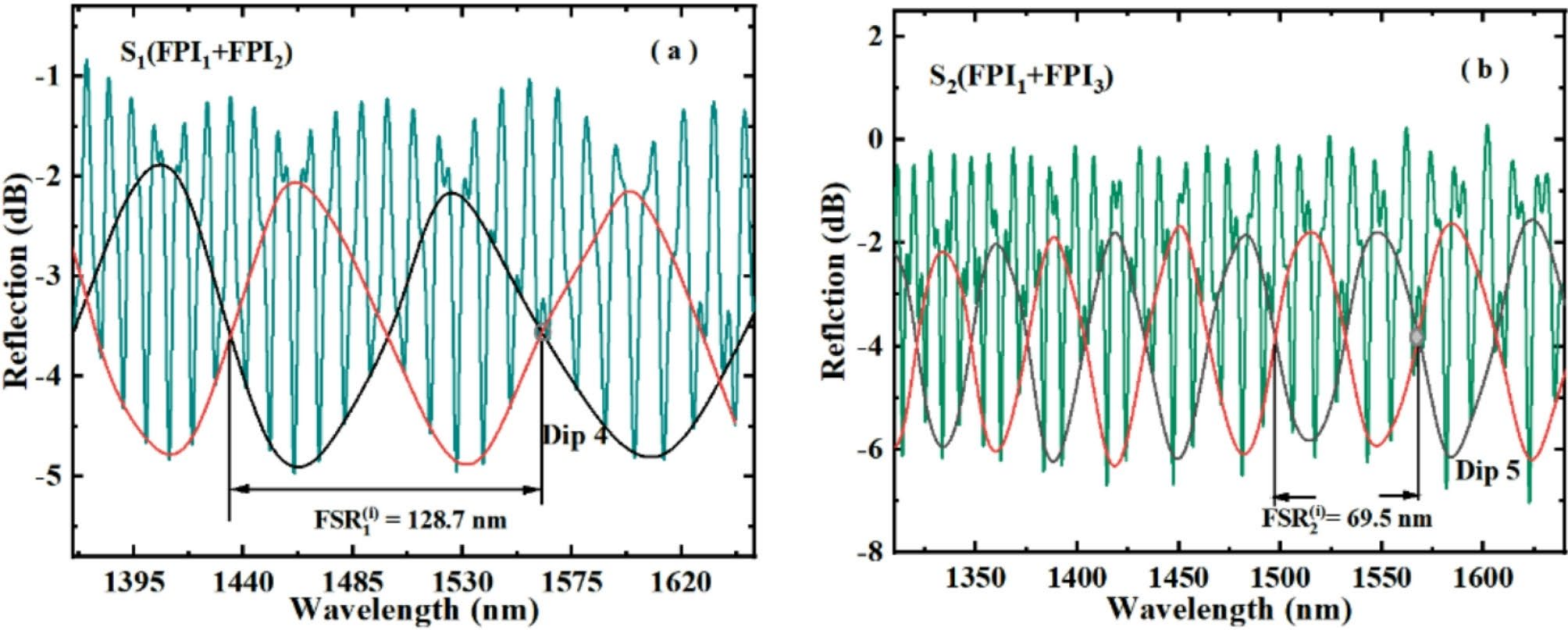
Spectral diagrams of (**a**) S_1_ and (**b**) S_2_.

Figure 5 illustrates the spectra of two HVE sensors S_1_ and S_2_ formed in their parallel. In Fig. 5, the wavelength and intensity of the spectrum are taken out one by one at every other sampling point, and then the smooth curve is drawn in a “B-spline” way to obtain the inner envelope of the spectrum, namely the red line and black line in the spectrum. In sensing measurements, because of the very wide envelope, it is sometimes impossible to accurately read the position of the spectrum to understand the accurate movement of the spectrum; However, the internal intersection can provide an accurate wavelength values. Therefore, Dip 4 and Dip 5 are selected as the observation points of S_1_ and S_2_ in the heating experiment. The $FSR_{{{in{\text{-}}en}}_{{1}}}^{1}$ and $FSR_{{{in{\text{-}}en}}_{{2}}}^{1}$ of the inner-envelope of S_1_ and S_2_ recorded in Fig. 5 are 128.7 nm and 69.5 nm, respectively. The FSR difference between the two sensors mainly comes from the detuning difference of the two FPIs that make up the sensor, which leads to different temperature sensitivity amplification factor. The experimental results also confirm this point. In the following temperature test, Dip 1, Dip 2, Dip 3, Dip 4 and Dip 5 are used to measure the environmental temperature, and their comparison was used to study the influence of structure in parallel on the sensitivity of the sensor.

## Experiment results and discussion

### Experimental device

**Fig. 6:**
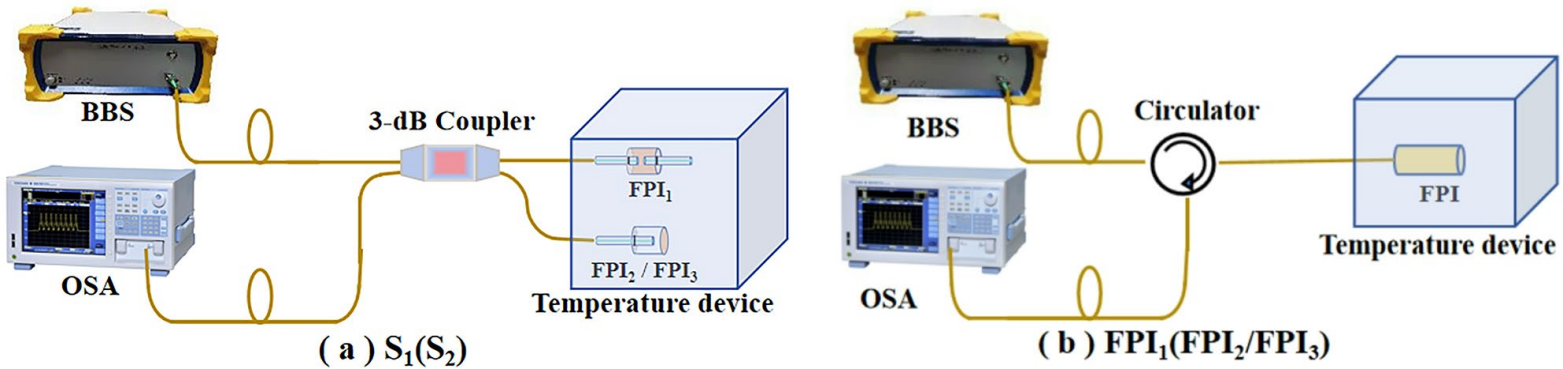
Schematic of the experimental device of (**a**) S_1_ (S_2_), and (**b**) FPI_1_ (FPI_2_/FPI_3_) measuring the temperature.

Figure 6a describes the schematic of the temperature experimental equipment of S_1_ and S_2_. In the temperature measurement of S_1_ or S_2_, low polarization ultra-wideband light source (BBS, FL-ASE) is used as the input light source, and OSA (AQ6370D) is used to record the spectrum of the sensor. The optical fiber 3-dB coupler is used to connect BBS, OSA and S_1_ (or S_2_), which is provided with a temperature change environment by a precise temperature and humidity chamber (WHTH-80 L, Dongguan, China) with an accuracy of 0.1 °C. In the temperature experiment, the humidity inside the test box is kept constant, and the temperature changes from 30 to 60 °C, with a change range of 1 °C or 5 ℃. After each measuring point was stabilized for 15 min, six groups of spectral data were recorded. Figure 6b displays the schematic diagram of single FPI_1_ or FPI_2_/FPI_3_ temperature experimental equipment. An optical circulator is used to connect BBS, OSA and FPI_1_ (or FPI_2_/FPI_3_), with the same temperature measurement method as S_1_ or S_2_.

### FPI_1_, FPI_2_, and FPI_3_ measuring temperature respectively

**Fig. 7:**
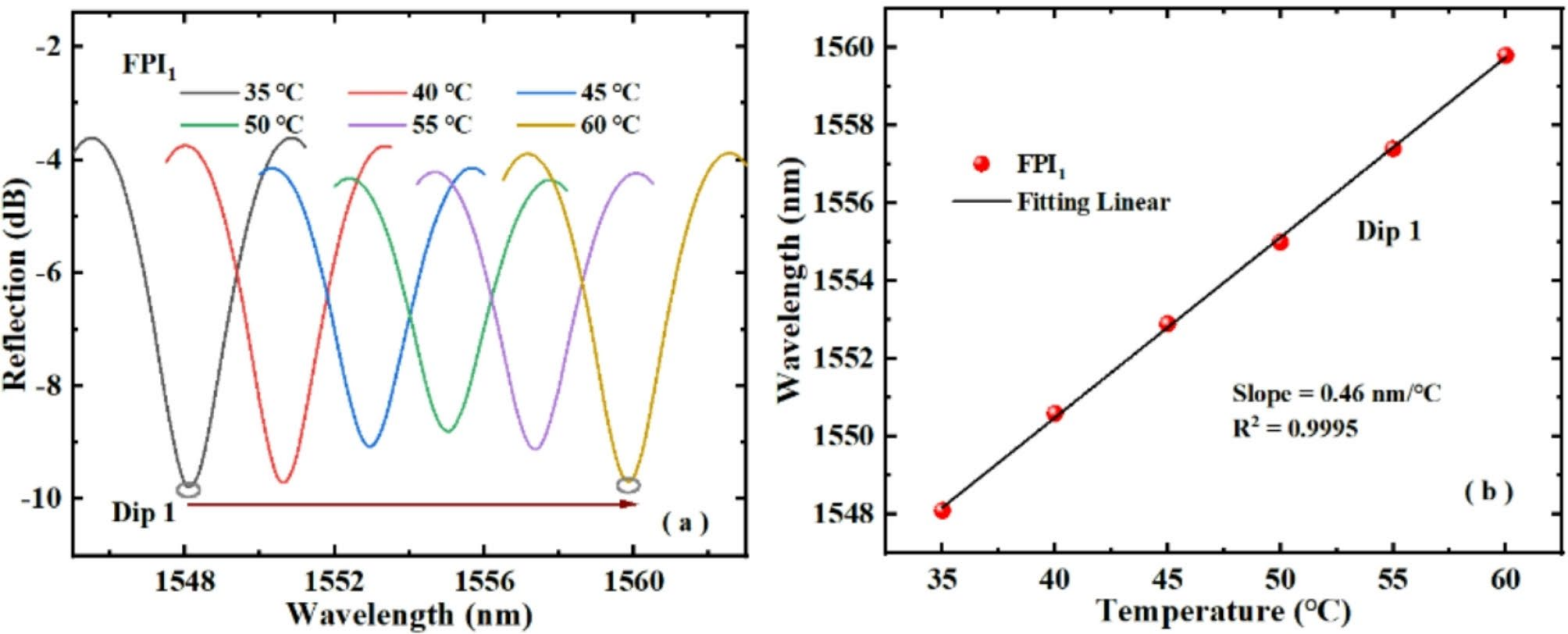
(**a**) Movement of Dip 1 wavelength of FPI_1_ with temperature rising, (**b**) fitting of the Dip 1 wavelength and temperature.

Firstly, we studied the temperature response of single FPI_1_, FPI_2_ and FPI_3_. Figure 7a presents how the Dip 1 of FPI_1_ changes with increasing temperature. As can be seen from Fig. 7a, with the rise of temperature, the Dip 1 shifts towards the long wavelength direction. Figure 7b displays the linear fitting of Dip 1 wavelength with temperature change. Evidently, the sensitivity of FPI_1_ is 0.46 nm/℃ with the rise of temperature. The experimental results confirm that FPI_1_ is sensitive to temperature, and the sensitivity sign is positive number.

**Fig. 8:**
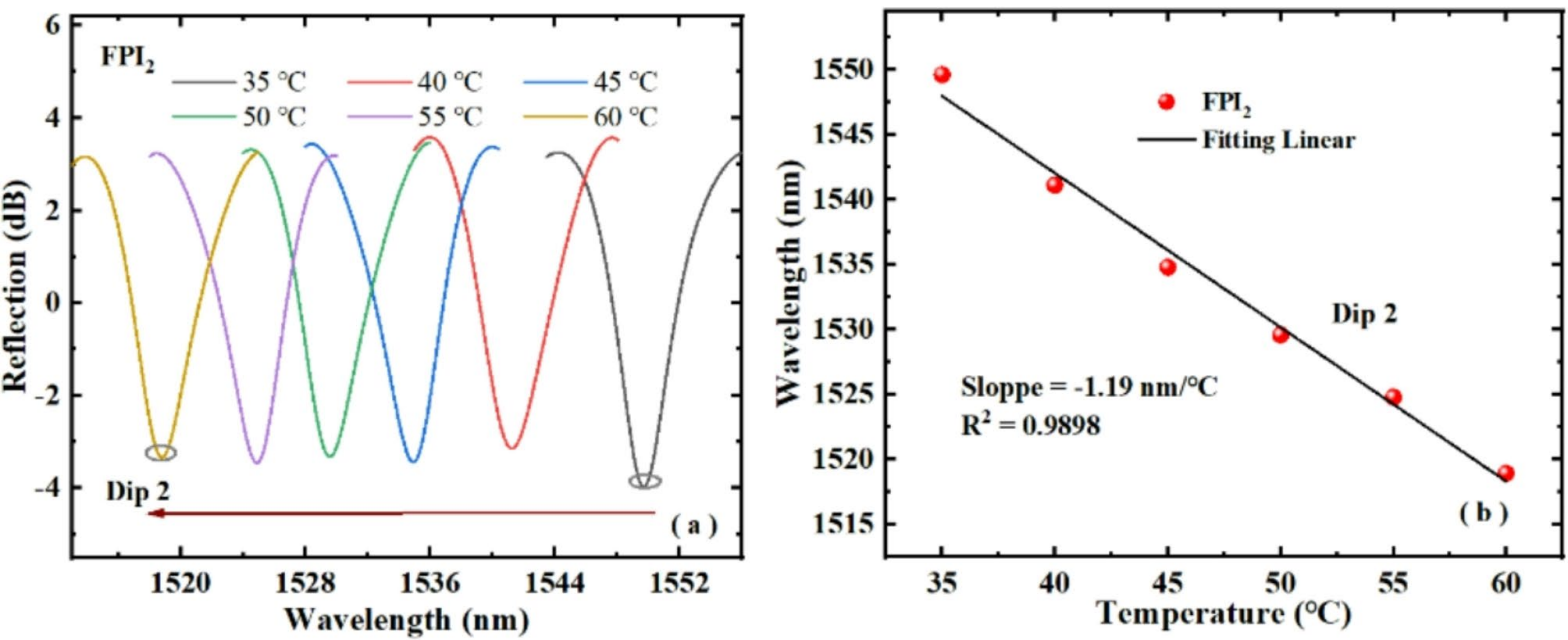
(**a**) Dip 2 wavelength changes of FPI_2_ with temperature rising, (**b**) fitting of the Dip 2 wavelength and temperature rise.

**Fig. 9:**
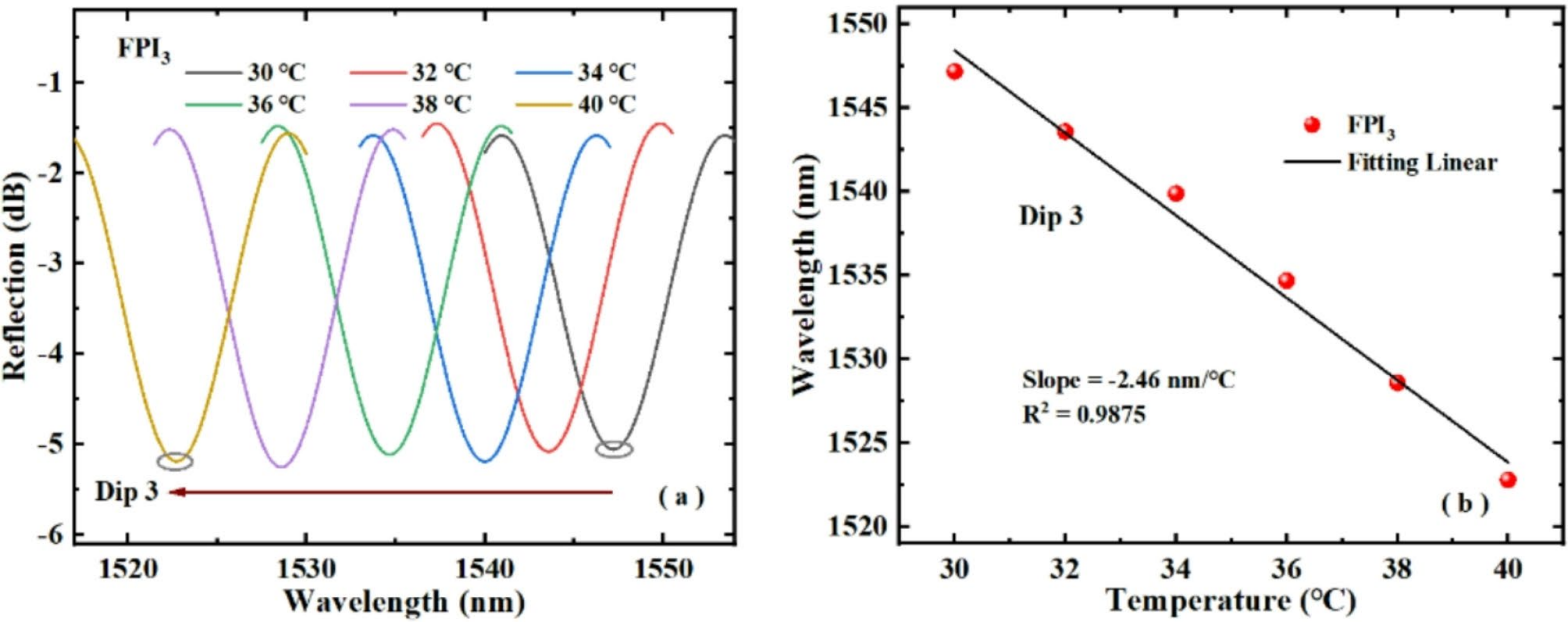
(**a**) Dip 3 wavelength changes of FPI_3_ with temperature rising, (**b**) fitting of the Dip 3 wavelength and temperature rise.

Figures 8 and 9 show the temperature responses of FPI_2_ and FPI_3_ respectively. From Figs. 8a and 9a, it can be seen that the Dip 2 and Dip 3 of FPI_2_ and FPI_3_ both drift towards the short wave direction with rising temperature. Figures 8b and 9b illustrates the linear fitting of the Dip 2 and Dip 3 with the change of temperature. Clearly, with the rise of temperature, the sensitivities of FPI_2_ and FPI_3_ are − 1.19 nm/℃ and − 2.46 nm/℃, respectively. The experimental results confirm that FPI_2_ and FPI_3_ are sensitive to temperature, and the sensitivity signs are negative number. Therefore, parallel connection of FPI_2_ or FPI_3_ and FPI_1_ can produce an enhanced Vernier effect.

### S_1_ (FPI_1_ + FPI_2_) measuring temperature

**Fig. 10:**
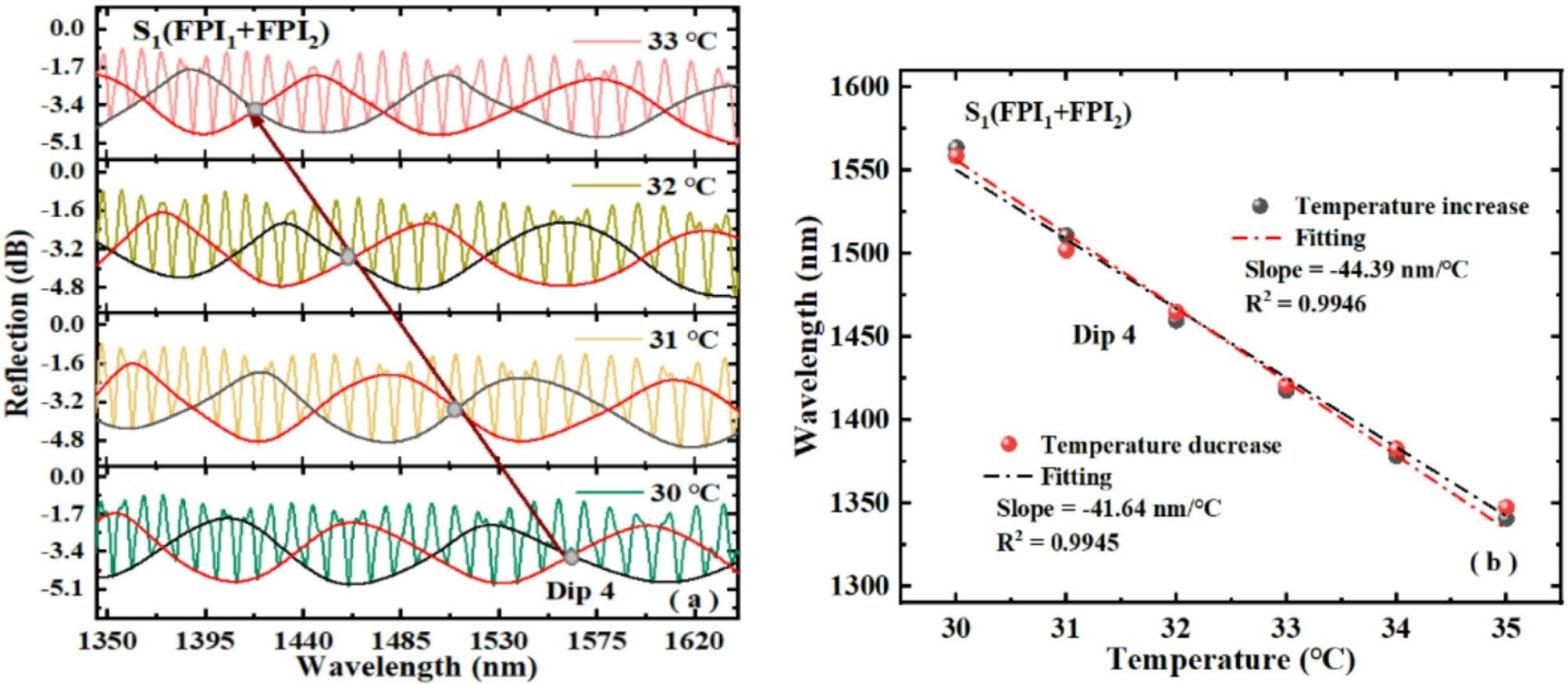
(**a**) Dip 4 wavelength changes of S_1_ as temperature rising, (**b**) fitting of the Dip 4 wavelength and temperature rise.

Next, the temperature sensitivity of S_1_ is measured by using the experimental device shown in Fig. 6a. In the S_1_′ temperature experiment, FPI_1_ and FPI_2_ are placed in a programmable constant temperature and humidity test box. The humidity in the experimental chamber is kept at 40%RH, and the temperature is measured starting from 30 ℃. When the temperature is stable, the data is recorded after 5 min. The measured value of each temperature are divided into three groups of records, and the average value is taken. The shift of the reflection spectrum of the sensor from 30 ℃ to 35 ℃ is recorded at intervals of 1℃. Figure 10 denotes the experimental results of S_1_ with respect to temperature. The arrow in Fig. 10a shows the movement of the internal intersection point Dip 4 with temperature. The FSR detuning ($\delta =2FS{R_1} - FS{R_2}$) of S_1_ is about − 1.2 nm. When the temperature rises, the spectrum of S_1_ shifts to a shorter wavelengths, which is contrary to the trend of FPI_1_ and consistent with that of FPI_2_, because the detuning of S_1_ is negative and FPI_2_ is positive. Figure 10b is a linear fitting of Dip 4 wavelength and temperature, from which it can be seen that the sensitivity of S_1_ to heating and cooling is − 44.39 nm/℃ and − 41.64 nm/℃, and the linear coefficients are 0.9946 and 0.9945 respectively. The experimental findings show that S_1_ has good linearity and the phenomenon of spectral drift is in good agreement with theory. We calculated the sensitivity of S_1_ using formula (12) to be − 47.6 nm/℃. The sensitivity of S_1_ obtained from the experiment is close to the sensitivity calculated using formula (12).

**Fig. 11:**
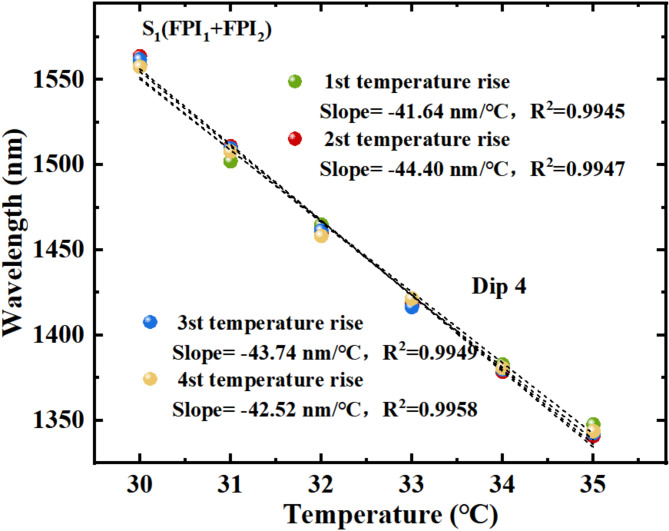
Four temperature measurement experimental data of Dip 4 wavelength.

To investigate the consistency of S_1_ in measuring temperature at different times, the repetitive experiment is conducted on S_1_. The temperature is measured by S_1_ once every half month to verify its performance. Figure 11 shows measured data obtained from four heating experiments of S_1_ under the same conditions. The sensitivities obtained from four heating experiments were − 41.64 nm/℃, − 44.40 nm/℃, − 43.74 nm/℃, and − 42.52 nm/℃, respectively, with R^2^ of 0.9945, 0.9947, 0.9949, and 0.9958. The average sensitivity is − 43.07 nm/℃. The experimental results exhibit that the data measured by four experiments (corresponding to Dip 4) are basically the same, which shows that the sensor has good measurement repeatability.

### S_2_ (FPI_1_ + FPI_3_) measuring temperature

**Fig. 12:**
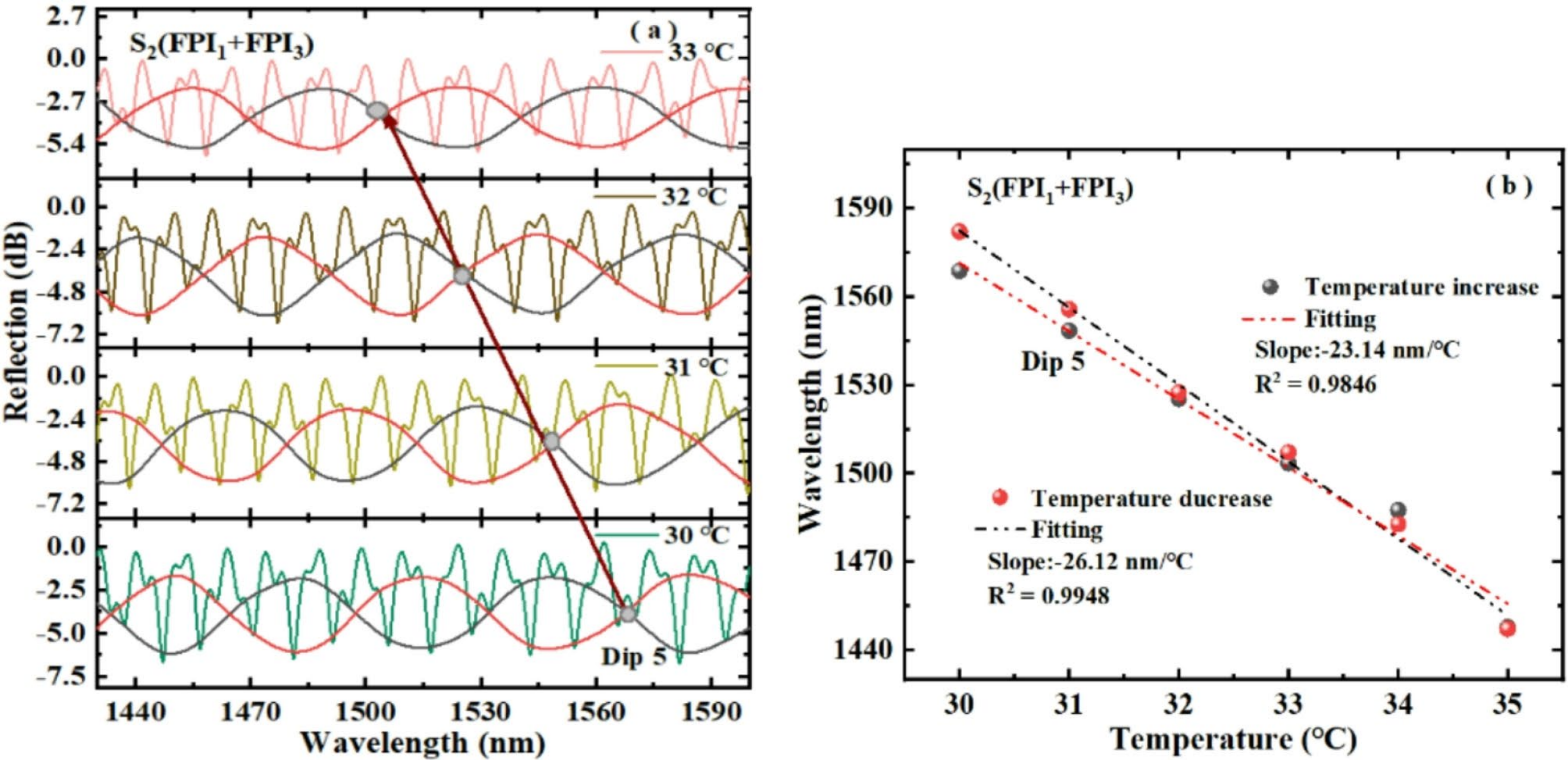
(**a**) Dip 5 wavelength changes of S_2_ with temperature rising, (**b**) fitting of the Dip 5 wavelength with temperature changes.

The temperature experiment of S_2_ is carried out according to the same measuring method of S_1_. The experimental results are shown in Fig. 12. The arrow in Fig. 12a denotes the movement of the internal intersection point Dip 5 of S_2_ with temperature. The FSR detuning ($\delta =2FS{R_1} - FS{R_3}$) of S_2_ is about − 1.6 nm, which is larger than the FSR detuning of S_1_, and the FSR detuning of S_2_ is also negative. Therefore, when the temperature rises, the reflection spectrum of S_2_ will also blue-shift, which is the same as the movement trend of FPI_3_. Figure 12b is a linear fitting of Dip 5 wavelength and temperature. Clearly, the sensitivity of temperature rise and temperature drop of S_2_ is − 23.14 nm/℃ and − 26.12 nm/℃, which is lower than that of S_1_, mainly because the internal envelope FSR of S_1_ is about twice that of S_2_. The linearity of S_2_ with the increase and decrease of temperature is 0.9846 and 0.9948, respectively. We calculated the sensitivity of S_2_ using formula (13) to be − 19.71 nm/℃. The sensitivity of S_2_ obtained from the experiment is close to the sensitivity calculated theoretically using formula (13).

**Fig. 13:**
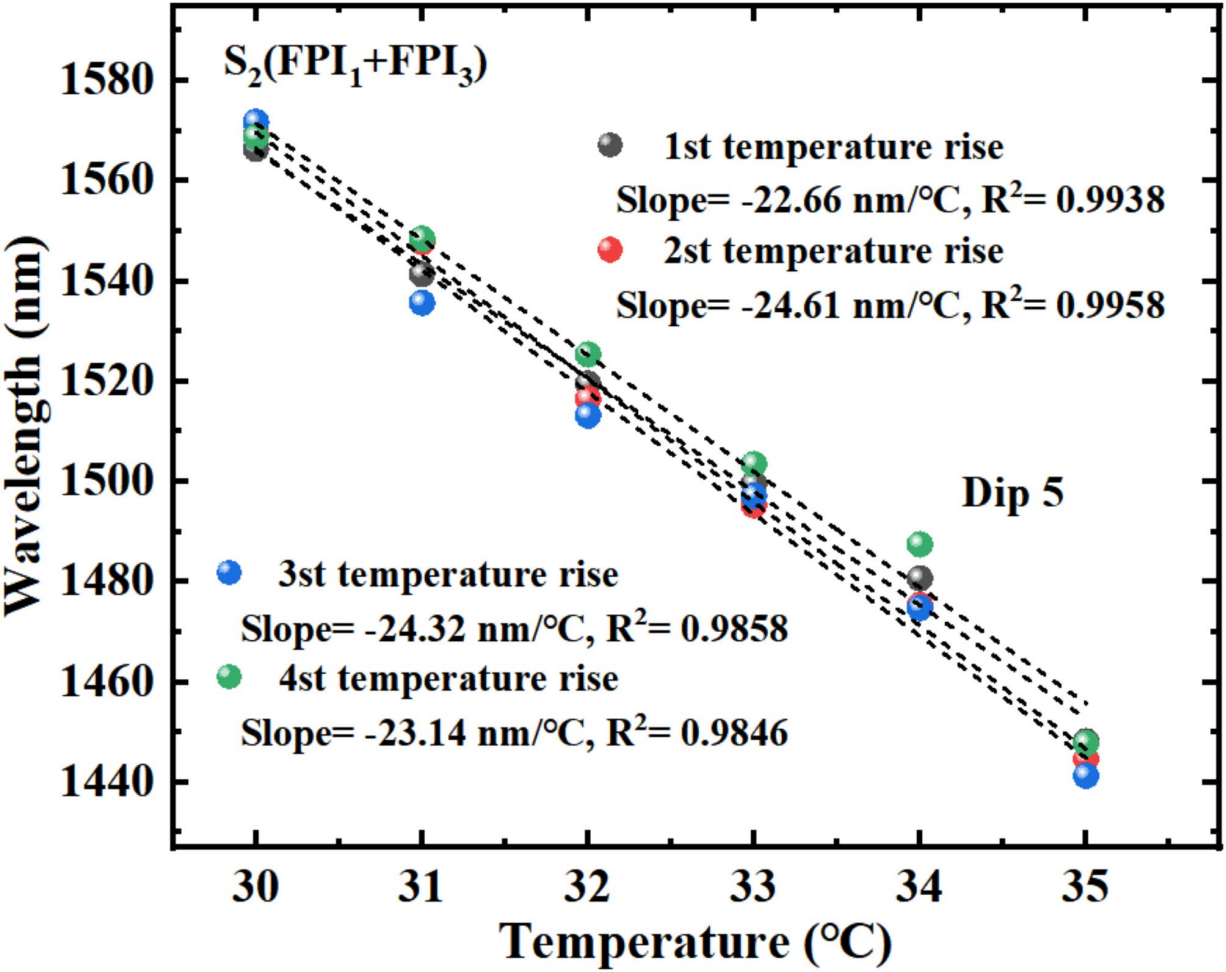
Four temperature changes experimental data of Dip 5 wavelength.

Figure 13 shows the measurement data obtained from four heating experiments of S_2_ under the same conditions. Obviously, the sensitivities obtained from four heating experiments were − 22.66 nm/℃, − 24.61 nm/℃, − 24.32 nm/℃, and − 23.14 nm/℃, respectively, with R^2^ of 0.9938, 0.9958, 0.9858, 0.9846. The average sensitivity is − 23.68 nm/℃. The experimental findings show that the data measured by four experiments (corresponding to Dip 5) are basically the same, which exhibits that the sensor has good measurement repeatability.

### Stability of FPI_1_, FPI_2_, FPI_3_, S_1_, and S_2_ measuring temperature

**Fig. 14:**
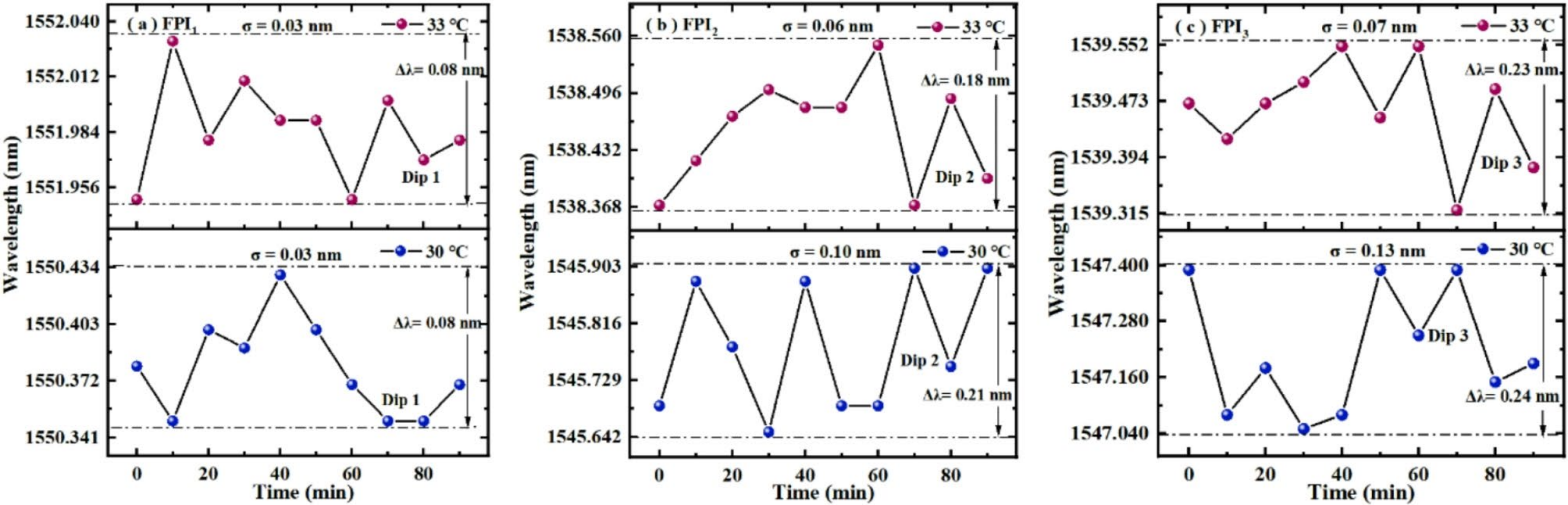
Stability experimental results of (**a**) FPI_1_, (**b**) FPI_2_, and (**c**) FPI_3_ measuring temperature.

Finally, we also conducted stability experiments on FPI_1_, FPI_2_, FPI_3_, S_1_, and S_2_ respectively. The specific experimental process is described as follows: the sensor is placed in the temperature experimental device, the temperature is set to be constant, and then the spectrum of the sensor is recorded every 10 min for 90 min. Finally, the long-term fluctuations of the sensor spectrum at a constant temperature were analyzed to determine its stability. First, when temperature is set to a constant value of 33 ℃ or 30℃, the long-term fluctuations of Dip 1 in FPI_1_, Dip 2 in FPI_2_, Dip 3 in FPI_3_ are recorded in Fig. 14.

In Fig. 14a, the standard deviation (SD) of Dip 1 wavelength fluctuation is very small, with values of 0.03 nm and 0.03 nm under two constant temperatures, respectively. This result indicates that FPI_1_ has better stability when measuring temperature. In addition, at two constant temperatures, the fluctuation range of Dip 1 wavelength is 0.08 nm and 0.08 nm, respectively, which translates to measurement maximum error of approximately 0.09 ℃. Indicating that the error caused the noise is lower, and the impact of noise on FPI_1_ is relatively small. In Fig. 14b, the SD of Dip 2 wavelength fluctuation is very small, with values of 0.06 nm and 0.10 nm under two constant temperatures, respectively. This result indicates that FPI_2_ has better stability when measuring temperature. In addition, at two constant temperatures, the fluctuation range of Dip 2 wavelength is 0.18 nm and 0.21 nm, respectively, which translates to measurement maximum error of approximately 0.09 ℃. Indicating that the error caused by the noise is lower, and the impact of noise on FPI_2_ is relatively small. In Fig. 14c, the SD of Dip 3 wavelength fluctuation is very small, with values of 0.07 nm and 0.13 nm under two constant temperatures, respectively. This result indicates that FPI_3_ has better stability when measuring temperature. In addition, at two constant temperatures, the fluctuation range of Dip 3 wavelength is 0.23 nm and 0.24 nm, respectively, which translates to measurement errors of approximately 0.05 ℃. Indicating that the error caused by the noise is lower, and the impact of noise on FPI_3_ is relatively small.

**Fig. 15:**
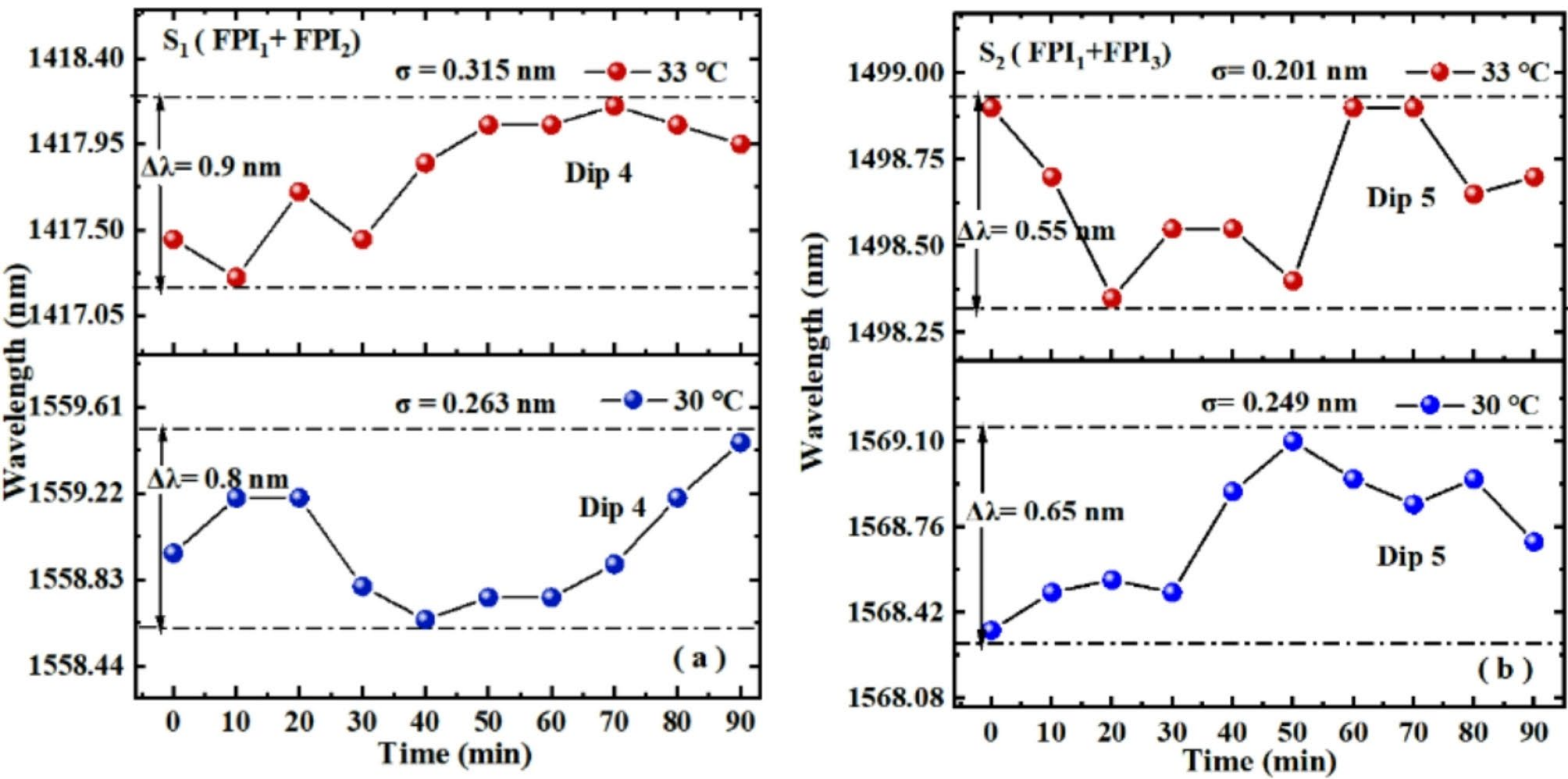
Stability experimental results of (**a**) S_1_ and (**b**) S_2_ measuring temperature.

When temperature is set to a constant value of 33 ℃ or 30℃, the long-term fluctuations of Dip 4 in S_1_ and Dip 5 in S_2_ are also recorded in Fig. 15. In Fig. 15a, the SD of Dip 4 wavelength fluctuation is small, with values of 0.315 nm and 0.263 nm under two constant temperatures, respectively. This result indicates that S_1_ has better stability when measuring temperature. In addition, at two constant temperatures, the fluctuation range of Dip 4 wavelength is 0.9 nm and 0.8 nm, respectively, which translates to measurement maximum error of approximately 0.01℃ (0.9/2/43.07 = 0.01). The experimental results show that the sensor has a small temperature measurement error. In Fig. 15b, the SD of Dip 5 wavelength fluctuation is small, with values of 0.201 nm and 0.249 nm under two constant temperatures, respectively. This result indicates that S_2_ has better stability when measuring temperature. In addition, at two constant temperatures, the fluctuation range of Dip 5 wavelength is 0.55 nm and 0.65 nm, respectively, which translates to measurement maximum error of approximately 0.014 ℃ (0.65/2/23.14 = 0.02). Indicating that the error caused the noise is lower, and the impact of noise on S_2_ is relatively small.

### Detection limit of FPI_1_, FPI_2_, FPI_3_, S_1_, and S_2_

We referred to the methods proposed in relevant literature^34–38^ to discuss whether the use of VE and HVE in sensors would affect their detection limits (DL). DL is the minimum value of environmental parameter accurately measured by sensor, and the DL of sensor based on spectral wavelength modulation is determined by the following formula^34–38^:

14 $$DL=\frac{R}{{\left| S \right|}}$$

where R is the wavelength resolution of the sensor and S is the sensitivity of the sensor. *R* can be expressed as follows^37,38^:

15 $$R=\sqrt {\sigma _{{_{{ampl{\text{-}}noise}}}}^{2}+\sigma _{{temp{\text{-}}induced}}^{2}+\sigma _{{spect{\text{-}}res}}^{2}}$$

where ${\sigma _{ampl{\text{-}}noise}}$ is the amplitude noise, ${\sigma _{temp{\text{-}}induced}}$ is the noise induced by temperature stabilization, ${\sigma _{spect{\text{-}}res}}$ is the standard deviation of spectral resolution. R is determined by these three noises.

In the stability experiment of the sensor, we obtained the SD of the sensor wavelength fluctuation under various noise effects at a constant temperature. Referring to the approximate processing methods provided in Refs^37,38^. , we use three times the maximum SD (*k* = 3 uncertainty) to approximate R. This approximation is the result of the combined effect of three types of noise. Therefore, the Eq. (14) can be simplified into the following equations^37,38^:

16 $$DL=\frac{{3\sigma }}{{\left| S \right|}}$$

where *σ* is the maximum SD of the sensor at a constant temperature.

Therefore, according to Eq. (16), we can estimate the DLs of FPI_1_, FPI_2_, FPI_3_, S_1_, and S_2_, respectively, and the results are shown in Table 1. According to the results in Table 1, it can be concluded that after using VE and HVE in the sensor, the corresponding DLs have also been improved due to the significant increase in sensitivity, by almost an order of magnitude.

**Table 1:** Comparison of the detection limits of FPI_1_, FPI_2_, FPI_3_, S_1_, and S_2_.

Sensor	Temperature sensitivity (S)	Maximum standard deviation (σ)	Detection limit (DL)
FPI_1_	0.46 nm/℃	0.03 nm	0.20 ℃
FPI_2_	− 1.19 nm/℃	0.10 nm	0.25 ℃
FPI_3_	− 2.46 nm/℃	0.13 nm	0.16 ℃
S_1_	− 44.39 nm/℃	0.315 nm	0.02 ℃
S_2_	− 23.14 nm/℃	0.249 nm	0.03 ℃

### Comparison with existing HVE temperature sensors

We have conducted a detailed comparison of the sensor structure, sensing materials, manufacturing methods, temperature sensitivity, and other aspects of the existing HVE structure temperature sensors. The results are shown in Table 2. The comparison results from Table 2 show that the proposed sensor structure is similar to most temperature sensor structures, using two FPIs in parallel. The two FPIs are easy to manufacture, low-cost, and easy to replicate. The sensitive material PDMS used in the structure has excellent thermal sensitivity. HVE is used to improve the sensitivity of the sensors. Moreover, the sensitivity of the proposed sensor is only lower than those in Refs^25,28,33^. , but much higher than those in other references. However, the use of SI structure in Ref^25^. requires a very long PMF. The sensors in Refs^28,33^. are relatively complex to manufacture. Therefore, the proposed sensor structure is simple, easy to manufacture, and has excellent performance, which has promotional value in industrial production.

**Table 2:** Comparison of the existing HVE temperature sensors.

Year	Sensor configuration	Sensing materials	Temperature sensitivity	References
2021	Two cascaded FPIs and HVE	PMF	3.66 nm/°C	^24^
2022	Two cascaded SIs and HVE	PMF	− 53.3 nm/°C	^25^
2023	Two cascaded FPIs and HVE	HCF/SMF	0.176 nm/°C	^26^
2023	Two cascaded FPIs and HVE	PDMS	− 3.4 nm/°C	^27^
2023	Two cascaded FPIs and HVE	PDMS	50.351 nm/°C	^28^
2023	Two cascaded FPIs and HVE	BHCF	0.298 nm/°C	^29^
2023	Two cascaded SIs and HVE	PMF	− 40.73 nm/℃	^30^
2023	FPI cascaded with MI and HVE	PDMS	− 19.22 nm/°C	^31^
2023	Two cascaded FPIs and HVE	SCF/HCF	0.1097 nm/℃	^32^
2024	Two parallel FPIs and HVE	PDMS	− 99.66 nm/°C	^33^
Now	Two parallel FPIs and HVE	PDMS	− 44.39 nm/℃	This work

## Conclusion

This article introduces a highly sensitive fiber optic temperature sensor based on enhanced HVE. The temperature sensor comprises two parallel temperature sensitive FPIs. By controlling the length of the FPI resonant cavity, the FSR of the two FPIs is approximately doubled. When the temperature rises, the interference spectra of two FPIs made of temperature sensitive material PDMS and ceramic ferrule are respectively red shifted and blue shifted, thus forming an enhanced HVE. Two enhanced HVE sensors S_1_ and S_2_ with different FSR detuning were prepared using this method in the article. The experimental findings illustrates that the temperature sensitivity of S_1_ and S_2_ are − 44.39 nm/℃ and − 23.14 nm/℃, respectively, which effectively improves the sensitivity compared to a single sensor. In addition, the sensor is reusable and has stable performance, which has very good application prospects.

## Data Availability

Data will be made available on request. If anyone wants to request data from this study, they should contact the corresponding author Prof. Chao Jiang, whose email is jiangchao1969@hbnu.edu.cn.
